# Can the Addition of NT-proBNP and Glucose Measurements Improve the Prognostication of High-Sensitivity Cardiac Troponin Measurements for Patients with Suspected Acute Coronary Syndrome?

**DOI:** 10.3390/jcdd8090106

**Published:** 2021-09-01

**Authors:** Peter A. Kavsak, Shawn E. Mondoux, Mark K. Hewitt, Craig Ainsworth, Stephen Hill, Andrew Worster

**Affiliations:** 1 Department of Pathology and Molecular Medicine, McMaster University, Hamilton, ON L8S 4L8, Canada; hills@mcmaster.ca; 2 Department of Medicine, Division of Emergency Medicine, McMaster University, Hamilton, ON L8S 4L8, Canada; shawn.e.mondoux@gmail.com (S.E.M.); mark.hewitt@medportal.ca (M.K.H.); worstea@mcmaster.ca (A.W.); 3 Department of Medicine, Division of Cardiology, McMaster University, Hamilton, ON L8S 4L8, Canada; ainswoc@mcmaster.ca

**Keywords:** high-sensitivity cardiac troponin, natriuretic peptides, glycemia, acute coronary syndrome, emergency department

## Abstract

Guidelines published in 2021 have supported natriuretic peptide (NP) testing for the prognostication in patients with acute coronary syndrome (ACS) and for the diagnosis of chronic and acute heart failure (HF). Our objective was to determine if the addition of N-terminal pro B-type NP (NT-proBNP) and glucose to high-sensitivity cardiac troponin (hs-cTn) could better identify emergency department (ED) patients with potential ACS at low- and high-risk for a serious cardiovascular outcome over the next 72 h. The presentation sample in two different ED cohorts which enrolled patients with symptoms suggestive of ACS within six hours of pain onset (Cohort-1, *n* = 126 and Cohort-2, *n* = 143) that had Abbott hs-cTnI, Roche hs-cTnT, NT-proBNP and glucose were evaluated for NT-proBNP alone and combined with hs-cTn and glucose for the primary outcome (composite which included death, myocardial infarction, HF, serious arrhythmia and refractory angina) via receiver-operating characteristic (ROC) curve analyses with area under the curve (AUC) and diagnostic estimates derived. The AUC for NT-proBNP for the primary outcome was 0.68 (95% confidence interval (CI): 0.59–0.76) and 0.75 (95%CI: 0.67–0.82) in Cohort-1 and 2, respectively, with the 125 ng/L cutoff yielding a higher sensitivity (≥75%) as compared to the 300 ng/L cutoff (≥58%). Using the 125 ng/L cutoff for NT-proBNP with the published glucose and hs-cTn cutoffs for risk-stratification produced a new score (GuIDER score for Glucose, Injury and Dysfunction in the Emergency-setting for cardiovascular-Risk) and yielded higher AUCs as compared to NT-proBNP (*p* < 0.05). GuIDER scores of 0 and 5 using either hs-cTnI/T yielded sensitivity estimates of 100% and specificity estimates > 92% for the primary outcome. A secondary analysis assessing MI alone in the overall population (combined Cohorts 1 and 2) also achieved 100% sensitivity for MI with a GuIDER cutoff ≥ 2, ruling-out 48% (Roche) and 38% (Abbott) of the population at presentation for MI. Additional studies are needed for the GuIDER score in both the acute and ambulatory setting to further refine the utility, however, the preliminary findings reported here may present a pathway forward for inclusion of NP testing for ruling-out serious cardiac events and MI in the emergency setting.

## 1. Introduction

Natriuretic peptide (NP) testing is of clinical value in the diagnosis of heart failure (HF) but also in patients with suspected acute coronary syndrome (ACS) [1,2]. The HF definition includes NP measurement interpretation and cutoffs for both ambulatory and acute settings. For HF, the N-terminal pro B-type NP (NT-proBNP) assay cutoff of ≥125 ng/L has been supported by other machine-learning based tools, further highlighting the role of NP testing using this cutoff [3]. Early decision-making and risk-stratification of suspected ACS using high-sensitivity cardiac troponin (hs-cTn) measurement has been reportedly improved with the incorporation of additional laboratory tests [1,4]. The clinical chemistry score (CCS) is an algorithm incorporating glucose measurement and the estimated glomerular filtration rate (eGFR) that has been demonstrated to improve the diagnostic and prognostic performance of hs-cTn in patients with possible ACS [4,5,6,7,8]. The glucose and hs-cTn cutoffs in the CCS have been validated with literature to support their use [9,10,11]. More controversial is the eGFR cutoff of 90 mL/min/1.73 m^2^ as the 60 mL/min/1.73 m^2^ cutoff has also been used to identify normal individuals [12]. Intriguingly, in patients with their measured GFR between ≥60 and <90 mL/min/1.73 m^2^ by either the iohexol plasma clearance or Cr^51^-EDTA plasma clearance methods the median concentration of NT-proBNP in this group (*n* = 148) was 121 ng/L [13]. As NT-proBNP is known to be cleared via the kidneys, using the 125 ng/L cutoff for NT-proBNP may serve not only as a marker of heart dysfunction but perhaps also as a surrogate of possibly reduced renal function [13]. Accordingly, we assessed the utility of NT-proBNP alone and in conjunction with glucose and hs-cTn using the ≥125 ng/L threshold (rather than eGFR) to determine its diagnostic performance for detecting a serious cardiovascular outcome in patients with suspected ACS.

## 2. Methods

### 2.1. Study Cohorts

**Figure 1:**
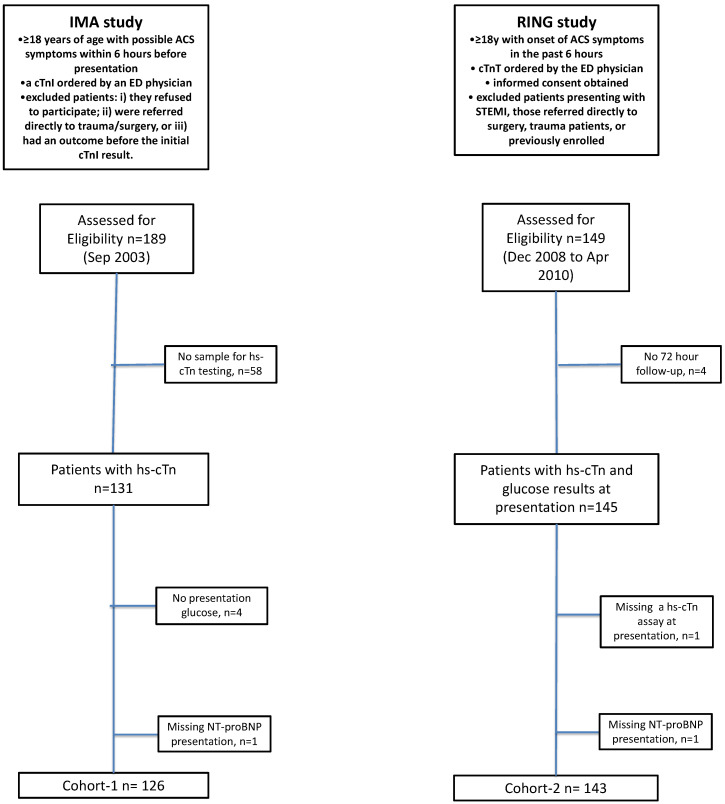
Flow diagram of cohorts used in this study.

We evaluated two different populations consisting of patients presenting with suspected ACS early after symptom-onset that had NT-proBNP measured in the presentation sample (Figure 1). In both cohorts, we obtained consent, blood for cardiac biomarker testing and demographic data. Details on storage and testing for NT-proBNP and the hs-cTn assays have been previously described [14,15]. Ethics approval was obtained by the Hamilton Health Sciences, McMaster University Research Ethics Board for Cohort-1 (#03-135) and the Hamilton Integrated Research Ethics Board (HiREB) for Cohort-2 (#08-427).

Briefly, Cohort-1 consisted of patients who consented to a study evaluating biomarkers for predicting serious cardiac outcomes (Research Ethics Board of Hamilton Health Sciences and McMaster University REB #03-135; the IMA study) [16]. In the month of September (2003), adult patients who presented with chest pain within 6 h of onset who consented to the study were enrolled. Eligibility criteria included: age 18 years or older, potential cardiac ischemia symptoms within 6 h from ED arrival and that the ED physician ordered cardiac troponin. Patients were excluded from the study if they were referred directly to the trauma or surgery teams; those in whom any of the study outcomes were obtained before the results of their first cardiac troponin results; patients who refused study participation or unable provide reliable contact information [14,16].

For Cohort-2 (from the RING study for Reducing the time Interval for identifying New Guideline defined MI in patients with suspected ACS in the ED; REB Project #08-427) [15,17] the design was similar as Cohort-1, with patient enrollment from December 2008 to April 2010. Specifically, patients who consented were included if: i. adult patient (>18 years of age) with onset of ACS symptoms within previous 6 h; ii. cardiac troponin ordered by ED physician; iii. consented to study and able to provide reliable contact for telephone follow up. Patients were excluded if: iv. ST elevation MI (STEMI); v. patient referred directly to surgery, or a trauma patient, or previous enrolment in RING study [15,17].

### 2.2. Outcomes

For both cohorts the outcomes were adjudicated with Cohort-1 using two physicians (emergency physician and a cardiologist) who independently evaluated all cases of suspected serious cardiac outcomes while blinded to cardiac biomarker data, with disagreements resolved by reaching a consensus opinion. Cohort-2 outcomes were also adjudicated by two physicians (ED physician and an internal medicine specialist) who independently adjudicated all outcomes and were blinded to the cardiac biomarker data. For both cohorts the composite outcome at 72 h (primary outcome) was used as previously described [14,15,16,17]. Briefly, for Cohort-1, the composite was death, MI, HF, serious arrhythmia and refractory ischemic cardiac pain (refractory angina), with the same outcomes used in Cohort-2 with the addition of revascularization (i.e., percutaneous intervention or coronary artery bypass graft), stroke and non-fatal cardiac arrest also included as part of the composite [18]. A secondary analysis was performed assessing MI alone in both cohorts.

### 2.3. Statistical Analysis

Data presented as counts, percentages, with non-parametric analyses used to describe distribution (i.e., median and interquartile range, IQR, with differences assessed via the Mann–Whitney U test and correlation by Spearman rho). Diagnostic estimates (benchmarks) included sensitivity (≥99%), specificity (≥90%), and positive (+LR ≥ 10) and negative (−LR ≤ 0.1) likelihood ratios as these estimates are not nearly as affected on prevalence as compared to predictive values [4,6,19]. For the calculation of the GuIDER score (Glucose, Injury and Dysfunction in the Emergency-setting for cardiovascular-Risk) the following algorithm was used: glucose ≥ 5.6 mmol/L = 1 point; NT-proBNP ≥ 125 ng/L = 1 point; with Abbott hs-cTnI concentration between 4–14 ng/L = 1 point/15–30 ng/L = 2 points/>30 ng/L = 3 points (Abbott GuIDER) and Roche hs-cTnT concentration between 8–18 ng/L = 1 point/19–30 ng/L = 2 points/>30 ng/L = 3 points (Roche GuIDER). The glucose and hs-cTn cutoffs were obtained from the CCS [4] with the 125 ng/L cutoff for NT-proBNP obtained from the proposed definition of HF and machine-based learning with population attributable risk percentage [2,3]. The GuIDER score ranged from 0 to 5 with the primary outcome (composite outcome) assessed based on each value. A secondary analysis consisted of assessing MI alone in the combined population designating the population into three groups: low-risk (GuIDER score 0 and 1), intermediate-risk (GuIDER score 2 and 3) and high-risk (GuIDER score 4 and 5) was also performed. ROC curve analyses were performed for NT-proBNP, hs-cTnI, hs-cTnT, Abbott GuIDER and Roche GuIDER with the DeLong test used to evaluate for differences between AUCs for each cohort for the primary outcome [20]. Analyses were performed using MedCalc^®^ Statistical Software version 20.009 (MedCalc Software Ltd., Ostend, Belgium; https://www.medcalc.org; 2021) with *p*-values < 0.05 considered significant.

## 3. Results

**Figure 2:**
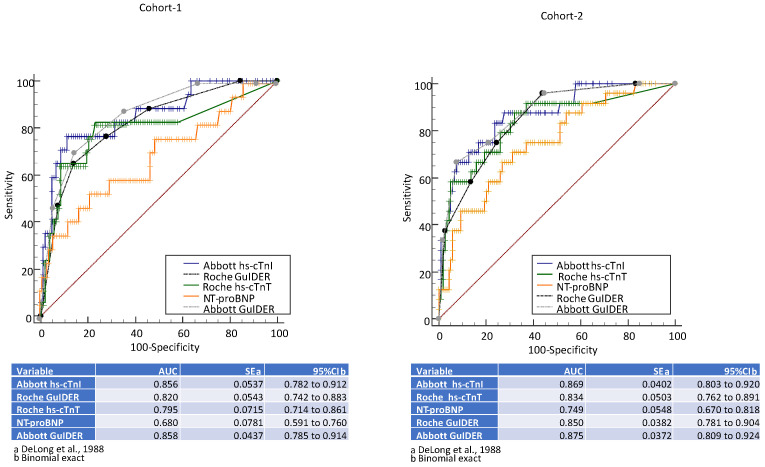
ROC curves for the primary composite outcome for the different biomarkers and algorithms.

There was no difference between the age of patients in Cohort-1 (*n* = 126, median (IQR) age = 59 years (49–72), age range = 23 to 93) versus Cohort-2 (*n* = 143, median (IQR) age = 60 years (49–70), age range = 24 to 90) (*p* = 0.78) with no difference between sexes in the cohorts (62% male in Cohort-1 versus 64% male in Cohort-2, *p* = 0.80) (Table 1). The number (percentage) of serious cardiac outcomes in Cohort-1 was 17 (13.5%) and 24 in Cohort-2 (16.8%) (*p* = 0.49). Pairwise comparison of ROC curves identified that there was a significant difference between AUCs for NT-proBNP versus the GuIDER scores in both cohorts (*p* < 0.05) (Figure 2). The AUCs for NT-proBNP were ≤ 0.75 with the 125 ng/L cutoff yielding a higher sensitivity (76% in Cohort-1 and 75% in Cohort-2) as compared to the 300 ng/L cutoff (59% in Cohort-1 and 58% in Cohort-2). The ≥5.6 mmol/L cutoff for glucose yielded a sensitivity of 88% in Cohort-1 and 83% in Cohort-2, with no correlation between glucose and NT-proBNP (rho = −0.04; *p* = 0.56).

Assessing the various GuIDER scores revealed that estimates of >99% sensitivity and >90% specificity were obtained in both cohorts, while only +LR and −LR exceeding the benchmarks in Cohort-2 (Table 2). Combining both cohorts and deriving the percentage of patients with and without serious cardiovascular outcomes indicated for Roche that 14% of the population would be low-risk (GuIDER score = 0) with none having serious outcomes. Conversely, 10% of the population would be labelled as high-risk (GuIDER score = 5) with 61% of this group having a serious cardiovascular outcome (Table 3). The same trends were observed for the Abbott GuIDER scores.

**Figure 3:**
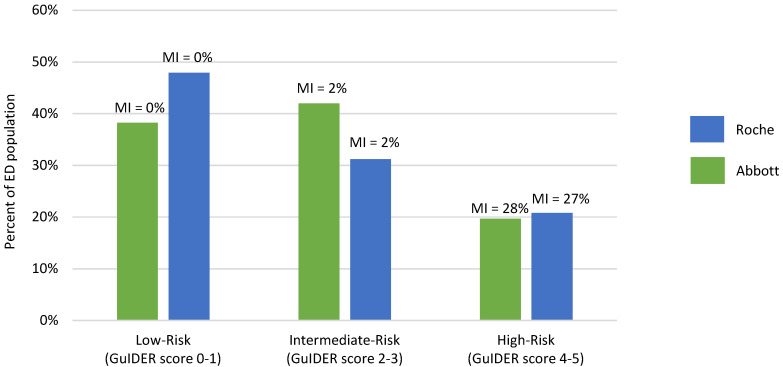
Percentage of ED population (both Cohort-1 and Cohort-2; *n* = 269) classified as low-risk (GuIDER score 0 and 1), intermediate-risk (GuIDER score 2 and 3) and high-risk (GuIDER score 4 and 5) and the prevalence of MI in these three groups.

Secondary analyses for MI alone in the overall population assessing the percentage of patients that could be further classified as low-risk (GuIDER score 0 and 1), intermediate-risk (GuIDER score 2 and 3) and high-risk (GuIDERscore 4 and 5) revealed a prevalence of MI of 0%, 2% and 27% (Roche)/28% (Abbott) in the respective groups (Figure 3). The percentage of the ED population that would be classified as low-risk was 48%, intermediate-risk 31% and high-risk 21% using Roche hs-cTnT (similar trend for Abbott hs-cTnI). The diagnostic estimates for MI using the GuIDER score ≥ 2 as the cutoff yielded a sensitivity of 100% (95%CI: 80.5–100) for both Roche and Abbott with a specificity of 51.2% (95%CI:44.8–57.5, Roche) and 40.9% (95%CI: 34.7–47.2, Abbott).

## 4. Discussion

The present analyses indicates that the ≥125 ng/L cutoff for NT-proBNP is helpful for increasing the sensitivity for detection of a serious cardiovascular event and can inform clinicians on an appropriate cutoff for assessing patients with possible ACS. The lack of correlation between NT-proBNP and glucose is similar to previous findings of no correlation between glucose and cardiac troponin [21]. Together, these findings strengthen the inclusion of glucose with NT-proBNP and cardiac troponin as each of the biomarkers provide independent information.

By including NT-proBNP (using the established cutoff of 125 ng/L), with cardiac troponin and glucose to form the GuIDER score; patients at presentation can be immediately identified as being low- and high-risk. Combining these biomarkers may also help in further risk stratification for patients without possible ACS as has been recently shown using machine-based learning algorithms [3]. However, the one advantage of the GuIDER score over other algorithms is the limited number of variables and calculations needed to obtain such scores.

Limitations in the present study include the following, despite the improved prognostic performance of the GuIDER score. First, the cohorts used (i.e., patients enrolled from 2003 in Cohort-1 and from 2008 to 2010 in Cohort-2) for the score estimates are not a contemporary ED population and may not be representative of the generalized ED presentation populations, especially across different geographic locations and countries. Second, the pragmatism of using the GuIDER score at presentation as ~75% of the population would lie between low- and high-risk for the primary outcome, and would require additional testing and investigations before patient disposition. However, these estimates are similar to other laboratory-based algorithms [4,22]. Moreover, if the GuIDER score is applied for MI only nearly half of the population (48% with the Roche GuIDER score) could be rule-out for MI at presentation. Third, long-term outcomes would further support the utility of the GuIDER score, as has been demonstrated for the CCS [4,5,6,7,8]. Fourth, the data are from two small cohorts with adjudication for MI performed with non-hs-cTn assays. However, this is not uncommon as many studies that have developed and assessed algorithms have used non-hs-cTn assays for MI adjudication [23,24]. Fifth, large, multicenter studies across different geographical locations are needed to further define the utility of the GuIDER score over hs-cTn alone, as has been demonstrated for the CCS [4,5,6,7,8,22].

In conclusion, future studies assessing the GuIDER score should include larger and more diverse patient populations (which is a weakness for the present analyses), patients with and without ACS symptoms, with long-term follow-up, and a possible head-to-head comparison between the validated CCS versus the GuIDER score.

## Figures and Tables

**Table 1:** Baseline characteristics of Cohort-1 and Cohort-2. Data presented as counts, percentages and median (IQR).

Variable	Cohort-1 (*n* = 126)	Cohort-2 (*n* = 143)
Age, Years	59 (49–72)	60 (49–70)
Sex, Male	78 (62%)	92 (64%)
Presenting with Chest Pain	107 (85%)	133 (93%)
Glucose, mmol/L	6.2 (5.4–7.4)	6.0 (5.2–7.8)
Glucose ≥ 5.6 mmol/L	88 (70%)	94 (66%)
NT-proBNP, ng/L	138 (38–504)	136 (33–325)
NT-proBNP ≥ 125 ng/L	67 (53%)	76 (53%)
hs-cTnT, ng/L	5 (<3–16)	7 (<3–17)
hs-cTnT > 14 ng/L	33 (26%)	44 (31%)
hs-cTnI, ng/L	7 (4–15)	2 (<1–10)
hs-cTnI > 26 ng/L	19 (15%)	23 (16%)

**Table 2:** Sensitivity, specificity and likelihood ratios for the GuIDER scores in Cohort-1 and Cohort-2 for primary outcome.

**Roche GuIDER Scores in Cohort-1 (AUC = 0.82)**								
**Criterion**	**Sensitivity**	**95% CI**	**Specificity**	**95% CI**	**+LR**	**95% CI**	**−LR**	**95% CI**
>0	100	80.5–100.0	15.6	9.4–23.8	1.18	1.1–1.3	0.00	
>1	88.2	63.6–98.5	54.1	44.3–63.7	1.92	1.5–2.5	0.22	0.06–0.8
>2	76.5	50.1–93.2	72.5	63.1–80.6	2.78	1.9–4.2	0.32	0.1–0.8
>3	64.7	38.3–85.8	86.2	78.3–92.1	4.70	2.6–8.5	0.41	0.2–0.8
>4	47.1	23.0–72.2	92.7	86.0–96.8	6.41	2.8–14.8	0.57	0.4–0.9
**Roche GuIDER Scores in Cohort-2 (AUC = 0.85)**								
**Criterion**	**Sensitivity**	**95% CI**	**Specificity**	**95% CI**	**+LR**	**95% CI**	**−LR**	**95% CI**
>0	100	85.8–100.0	16.8	10.6–24.8	1.20	1.1–1.3	0.00	
>1	95.8	78.9–99.9	56.3	46.9–65.4	2.19	1.8–2.7	0.074	0.01–0.5
>2	75.0	53.3–90.2	75.6	66.9–83.0	3.08	2.1–4.6	0.33	0.2–0.7
>3	58.3	36.6–77.9	86.6	79.1–92.1	4.34	2.5–7.7	0.48	0.3–0.8
>4	37.5	18.8–59.4	97.5	92.8–99.5	14.87	4.3–50.9	0.64	0.5–0.9
**Abbott GuIDER Scores in Cohort-1 (AUC = 0.86)**								
**Criterion**	**Sensitivity**	**95% CI**	**Specificity**	**95% CI**	**+LR**	**95% CI**	**−LR**	**95% CI**
>1	100	80.5–100.0	33.0	24.3–42.7	1.49	1.3–1.7	0.00	
>2	88.2	63.6–98.5	64.2	54.5–73.2	2.47	1.8–3.3	0.18	0.05–0.7
>3	70.6	44.0–89.7	85.3	77.3–91.4	4.81	2.8–8.3	0.34	0.2–0.7
>4	47.1	23.0–72.2	94.5	88.4–98.0	8.55	3.4–21.6	0.56	0.4–0.9
**Abbott GuIDER Scores in Cohort-2 (AUC = 0.88)**								
**Criterion**	**Sensitivity**	**95% CI**	**Specificity**	**95% CI**	**+LR**	**95% CI**	**−LR**	**95% CI**
>0	100	85.8–100.0	15.1	9.2–22.8	1.18	1.1–1.3	0.00	
>1	95.8	78.9–99.9	55.5	46.1–64.6	2.15	1.7–2.7	0.075	0.01–0.5
>2	75.0	53.3–90.2	79.0	70.6–85.9	3.57	2.4–5.4	0.32	0.2–0.6
>3	66.7	44.7–84.4	92.4	86.1–96.5	8.81	4.4–17.6	0.36	0.2–0.6
>4	33.3	15.6–55.3	98.3	94.1–99.8	19.83	4.5–87.7	0.68	0.5–0.9

**Table 3:** Percentage of primary outcome per the GuIDER scores using both Abbott hs-cTnI and Roche hs-cTnT for the combined cohorts.

**Abbott GuIDER Score**	**0**	**1**	**2**	**3**	**4**	**5**
						
Outcome	0	1	7	5	12	16
No Outcome	27	75	62	39	17	8
% Outcome	0%	1%	10%	11%	41%	67%
n (total = 269)	27	76	69	44	29	24
% of Patients	10%	28%	26%	16%	11%	9%
Chi-Square for Trend *p* < 0.001						
**Roche GuIDER Score**	**0**	**1**	**2**	**3**	**4**	**5**
Outcome	0	3	7	6	8	17
No Outcome	37	89	43	28	20	11
% Outcome	0%	3%	14%	18%	29%	61%
n (total = 269)	37	92	50	34	28	28
% of Patients	14%	34%	19%	13%	10%	10%
Chi-Square for Trend *p* < 0.001						

## Data Availability

The studies were conducted before data sharing processes were in place, and thus individual data are not available. The data are not publicly available due to privacy.
